# Multi-Omics Integration Reveals Short and Long-Term Effects of Gestational Hypoxia on the Heart Development

**DOI:** 10.3390/cells8121608

**Published:** 2019-12-11

**Authors:** Yu Gao, Chiranjib Dasgupta, Lei Huang, Rui Song, Ziwei Zhang, Lubo Zhang

**Affiliations:** 1 Department of Pharmaceutical Sciences, College of Pharmacy, University of Illinois Chicago, Chicago, IL 60612, USA; zzhan60@uic.edu; 2 Lawrence D. Longo, MD Center for Perinatal Biology, Department of Basic Sciences, Loma Linda University School of Medicine, Loma Linda, CA 92350, USA; cdasgupta@llu.edu (C.D.); lehuang@llu.edu (L.H.); rsong01@llu.edu (R.S.)

**Keywords:** hypoxia, heart, proteome, metabolome, multi-omics integration

## Abstract

Antenatal hypoxia caused epigenetic reprogramming of methylome and transcriptome in the developing heart and increased the risk of heart disease later in life. Herein, we investigated the impact of gestational hypoxia in proteome and metabolome in the hearts of fetus and adult offspring. Pregnant rats were treated with normoxia or hypoxia (10.5% O_2_) from day 15 to 21 of gestation. Hearts were isolated from near-term fetuses and 5 month-old offspring, and proteomics and metabolomics profiling was determined. The data demonstrated that antenatal hypoxia altered proteomics and metabolomics profiling in the heart, impacting energy metabolism, lipid metabolism, oxidative stress, and inflammation-related pathways in a developmental and sex dependent manner. Of importance, integrating multi-omics data of transcriptomics, proteomics, and metabolomics profiling revealed reprogramming of the mitochondrion, especially in two clusters: (a) the cluster associated with “mitochondrial translation”/“aminoacyl t-RNA biosynthesis”/“one-carbon pool of folate”/“DNA methylation”; and (b) the cluster with “mitochondrion”/“TCA cycle and respiratory electron transfer”/“acyl-CoA dehydrogenase”/“oxidative phosphorylation”/“complex I”/“troponin myosin cardiac complex”. Our study provides a powerful means of multi-omics data integration and reveals new insights into phenotypic reprogramming of the mitochondrion in the developing heart by fetal hypoxia, contributing to an increase in the heart vulnerability to disease later in life.

## 1. Introduction

Many risk factors contribute to the development of heart disease that is a leading cause of death in the adult. Large studies including both epidemiological and animal studies indicate that an adverse intrauterine environment and fetal stress play a critical role in an increased risk of ischemic heart disease in adulthood [1,2,3,4,5]. A common form of fetal stress during gestation is hypoxia. Various conditions can cause prolonged fetal stress of intrauterine hypoxia, including preeclampsia and placental insufficiency, pregnancy at high altitude, maternal anemia, and heart and lung disease, among others. Previous studies have suggested that antenatal hypoxia increases the risk of cardiovascular disease and heightened heart vulnerability to ischemia and reperfusion injury later in life [6,7,8,9,10,11,12,13,14,15,16,17,18,19,20,21,22,23,24,25]. 

The mechanisms underlying developmental programming of the heart with ischemic-sensitive phenotype remain largely elusive. Previously, we demonstrated that fetal hypoxia caused gene hypermethylation and repression of protein kinase C epsilon and glucocorticoid receptors in the heart, which contributed to an increase in the susceptibility of the heart to ischemia and reperfusion injury in offspring [21,23,24]. More recently, we demonstrated that fetal hypoxia resulted in a global change in methylome and epigenomic reprogramming of the transcriptome in the developing heart in a developmental and sex dependent manner [26].

The effect of gestational hypoxia on an epigenotype exerts its influence on developmental plasticity by changing protein expression patterns and metabolites that reveal functional readouts of cellular activity and physiological status, and serve as surrogates to the phenotype. Herein, we conducted a follow-up study to investigate the impact of antenatal hypoxia on proteome and metabolome in the same tissues of fetal and adult offspring hearts that were investigated in the previous study [26]. We examined the short-term and long-term effects of gestational hypoxia on transcriptomics, proteomics, and metabolomics profiling in the heart. Of importance, by integrating multi-omics data in the same tissues, we were able to cross-validate the findings at different expression levels, and to identify key gene/protein/metabolite modules from the large interaction network that would have been missed by any single omics analysis. The unbiased assessment of multi-omics integration across the transcriptome, proteome, and metabolome revealed the major effect of antenatal hypoxia on the mitochondrion in the heart, especially in two clusters: (a) the first cluster associated with “mitochondrial translation”/“aminoacyl t-RNA biosynthesis”/“one-carbon pool of folate”/“DNA methylation”; and (b) the second cluster with “mitochondrion”/“TCA cycle and respiratory electron transfer”/“acyl-CoA dehydrogenase”/“oxidative phosphorylation”/“complex I”/“troponin myosin cardiac complex”. Thus, the present study reveals new insights into phenotypic reprogramming of the mitochondrion in the developing heart in response to the fetal stress of intrauterine hypoxia, and sheds light on the mechanisms underlying fetal programming of heart disease in adulthood.

## 2. Materials and Methods

### 2.1. Experimental Animals

Time-dated pregnant Sprague–Dawley (SD) rats were purchased from Charles River Laboratories (Portage, MI, USA) and were treated with hypoxia, as described previously [26]. Briefly, pregnant rats from day 15 to day 21 of gestation were treated with either normoxia of room air or hypoxia of 10.5% O_2_. After the treatment, hearts were isolated from near-term (21-day) fetuses and 5 month-old offspring that had been killed by decapitation under isoflurane anesthesia (5% inhalant in room air). The whole fetal heart and the left ventricle of adult offspring were used in the analyses. All experimental procedures and protocols were approved by the Institutional Animal Care and Use Committee of Loma Linda University and followed the guidelines in the National Institutes of Health Guide for the Care and Use of Laboratory Animals (IACUC#: 8190021; approval date: 7 August 2019).

### 2.2. Proteomics Analysis

Proteomic analysis was performed with TMT labelled total cardiac protein, followed by LC-MS/MS analysis on an LTQ-Orbitrap-Fusion instrument, as previously described [27]. Trypsin digested peptides and alkylated proteins were labeled with TMT Sixplex reagent set (ThermoFisher Scientific, Waltham, MA, USA). TMT labeling was performed according to the manufacturer’s instructions. Peptide sample representing each treatment group was TMT-labeled with one of the respective reporters at *m*/*z* = 126.1 (control female heart), 127.1 (hypoxic female heart), 128.1 (control male heart), 129.1 (hypoxic male heart), 130.1 (control fetal heart) and 131.1 (hypoxic fetal heart) were pooled. Five such pools (biological replicates) were used in this study. Each TMT-labeled protein pool were fractionated by strong cation-exchange (SCX) chromatography (Poly LC) into four fractions, namely (a) flow-through (FT), (b) 50 mM KCl eluent (E), (c) 200 mM KCl eluent (H), and (d) 5% ammonium hydroxide eluent (J), all in 20% acetonitrile (ACN); then desalted with a C18/hypercarb mixed resin Toptip column (Poly LC) using 60% ACN. Desalted TMT-labeled peptide fractions were next fractionated on a Multidimensional Protein Identification Technology (MudPIT)-2D-nanoLC/MS/MS platform. A two-dimension nanoAcquity UPLC (Waters, MA, USA) and an Orbitrap Fusion MS (ThermoFisher Scientific,) were coupled together for this purpose. 2D-nanoLC was configured with nanoAcquity UPLC, using two mobile phases: 20 mM ammonium formate (pH 10) and 100% ACN. Online fractionation was achieved by 5 min elution off a NanoEase trap column (PN# 186003682, Waters) using stepwise-increased concentration of ACN. A total of five fractions were generated with 13%, 18%, 21.5%, 27%, 50%, and 80% of ACN, respectively. The second dimension nano-UPLC method was also performed as described previously [28]. NanoLC/MS/MS was carried out with a data-dependent acquisition survey method. The acquisition time was set from 1–70 min. Nano ESI source was used with spray voltage at 2600 V, sweep gas at 0, and ion transfer tube temperature at 275 °C. For MS2 scan, Orbitrap mass analyzer was used using an auto/normal mode with resolution set at 30,000. Only precursor ions with intensity 50,000 or higher were selected for MS2 scan. High energy collision induced dissociation (HCD) was used for fragmentation activation using 33% normalized activation energy. MS2 mass range was set auto/normal with first mass set at 120 m/z. Only charge states from 2 to 6 were allowed for MS2 scan. All MS2 spectra were recorded in the profile mode. The raw MS files were processed and analyzed using ProluCID search engine with a custom built protein reference database constructed from combined RNAseq data of the rats [29]. Protein identification result was filtered at protein-level false discovery rate (FDR) of 1%. TMT reporter ion intensity was quantified using Census 2 [30]. 

### 2.3. Metabolomics Analysis

Global metabolic profiling of the heart samples was performed by Metabolon, Inc. (Durham, NC, USA). Briefly, after a series of organic and aqueous extractions and QA/QC, samples were analyzed by Ultrahigh Performance Liquid Chromatography-Tandem Mass Spectroscopy (UPLC-MS/MS) platform. By comparison to a library of authenticated standards that contain the mass to charge ratio (*m*/*z*), retention time/index (RI), and chromatographic data including MS/MS spectral data, compounds were identified. In addition, three criteria of the MS/MS forward and reverse scores between the experimental data and authentic standards, accurate mass match to the library +/- 10 ppm, retention index within a narrow RI window of the proposed identification were used for biochemical identifications. The MS/MS scores are based on a comparison of the ions present in the library spectrum to the ions present in the experimental spectrum. The use of all three data points to distinguish and differentiate biochemical minimized the potential limitation of similarities between these molecules based on one of these factors. The Metabolon Laboratory Information Management System (LIMS) contains more than 3300 commercially available standard compounds that are used for sample analysis on all platforms for determination of their analytical characteristics. Structurally unnamed biochemicals were identified in additional mass spectral entries by virtue of their recurrent nature (both chromatographic and mass spectral), which may be identified by future acquisition of a matching purified standard or by classical structural analysis. 

### 2.4. Bioinformatics and Statistics

All statistical analysis was conducted using our custom-built Python and R scripts (Python 2.7, Python Software Foundation, available online: https://www.python.org/download/releases/2.7/license/); R 3.5, The R Foundation, Vienna, Austria). Default parameters were used for all analyses unless otherwise mentioned. For K-mean clustering, number of clusters was determined using silhouette analysis. Integration of multi-omics data were performed using Fisher’s combined probability test using the sum of -2log(pi) to calculate a combined probability for each pathway. For t-Distributed Stochastic Neighbor Embedding (t-SNE), perplexity was set to 20 and the first 50 PCA components were used with exaggeration of 4. The analysis was repeated 15 times with different random seeds to ensure a stable clustering pattern. Quantitative proteomics data were normalized for each LC-MS experiment assuming equal total ion counts (TIC).

## 3. Results

### 3.1. Study Design

Pregnant rats were exposed to either normoxia (control group) or hypoxia (10.5% O_2_) for six days during gestational day 15 to day 21, as reported previously [26]. Supplementary Figure S1 shows the study design in detail. The fetal hearts were collected at day 21 of gestation, and the adult male and female offspring hearts were collected at five months after birth. The left ventricle of the adult heart and the whole fetal heart were used in the analyses of proteome and metabolome.

### 3.2. Effect of Antenatal Hypoxia on Proteome in the Heart

Quantitative proteomics data were obtained using TMT labeling following previously published procedures [27,30]. In total, we have identified 4881 proteins with protein level FDR < 0.01. We then filtered out all the singlet protein entries (i.e., protein with single unique peptide identification) resulting in a list of 3887 proteins. Within these data, after normalization we were able to reliably quantify 1537 proteins across all biological samples and conditions (Supplementary Data 1). 

**Figure 1:**
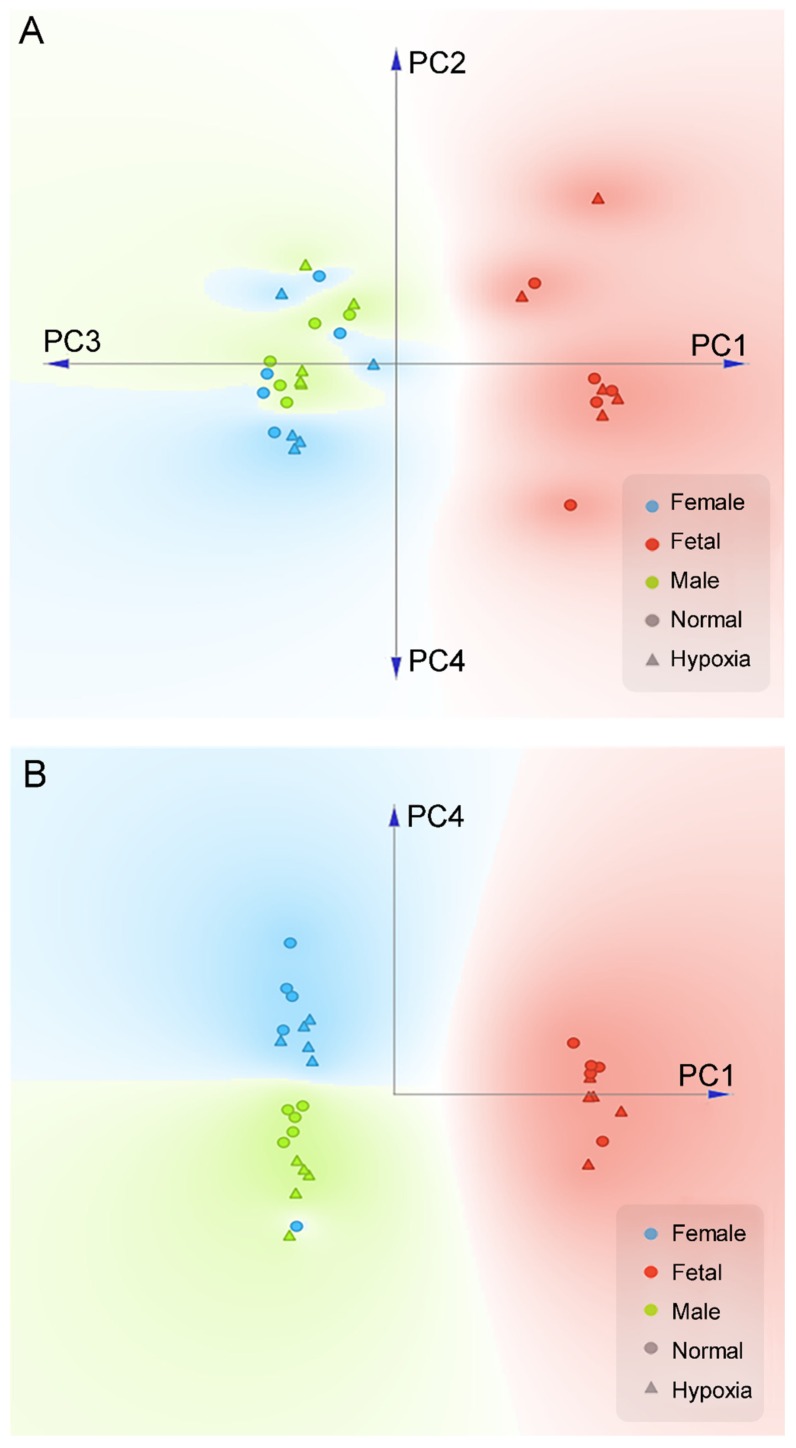
PCA plot of proteomics data in the heart. Pregnant rats were treated with normoxia or hypoxia (10.5% O_2_) from day 15 to day 21 of gestation. Proteomics profiling was determined in hearts isolated from near-term (21-day) fetuses and 5 month-old male and female offspring. (**A**) Linear projection of PC1 to PC4 of PCA shows fetal heart are significantly different than adult. (**B**) Linear projection of PC1 and PC4 shows that all but one sample could be cleanly separated by gender using only two principle components (PC1, PC4). Circle: normal; Triangle: hypoxia; Red: fetal; Blue: adult female; Green: adult male.

We first performed a PCA analysis to differentiate the gender effects from all experimental conditions. As shown in Figure 1A, when the first four components (72.7% variance covered) of PCA were projected onto a two-dimensional plane, we can clearly see a separation between the fetal samples (both hypoxia and normal conditions) and the adult samples. Within the first four components of PCA, PC1, and PC4 were able to cleanly separate gender effects for all but one samples (Figure 1B). This analysis showed that in addition to the development of fetus to adult, gender was a significant factor for differential proteome in the heart. From the first four PCA components, normoxic control and hypoxic groups cannot be clearly separated. Interestingly, we noticed that within each gender, it is possible to approximately separate the control and hypoxic conditions along the PC4 axis (Figure 1B). Therefore, we performed a K-mean clustering analysis on the PCA components to identify potential genes that differentiate hypoxic and control conditions in both genders. In total, we have identified 73 genes that have significant contributions to PC4 (Supplementary Figure S2).

We then performed functional network enrichment analysis using the STRING database (string-db.org). To improve network coverage, we used both human and mouse orthologues for this analysis as there were not enough annotations for rats. All the other pathway analyses were performed using rat data unless otherwise mentioned. We compared analysis results of functional network enrichment analysis using either mouse or human orthologues, and found no significant differences in the overall identified genes. (Supplementary Figure S21) The most significantly enriched pathways in biological processes are “cellular response to stress”, “proteolysis involved in cellular protein catabolic process” and “proteasome-mediated ubiquitin-dependent protein catabolic process” (Supplementary Figure S22). Within the “cellular response to stress” pathway, we have identified 20 genes from our data, consisting of three main groups: (1) the heat shock protein family (Dnajc3 (also known as Hsp40), Hspa5, Hsph1, Hspa8); (2) the proteasome complex and associated proteins (Psmd1, Psma3, Psmc4, Psmb7, Usp14, Usp5); and (3) the sarcoplasmic reticulum related proteins (Apoa4, Asna1, Bnip1, Chp1, Fkbp3, Hspa5, Kif1c, Map2k1, Pcyt1a, Rtn1, Rtn3, Sar1a, Surf4, Vapa, Vcp) (Supplementary Figure S23). These data showed a strong correlation. 

**Figure 2:**
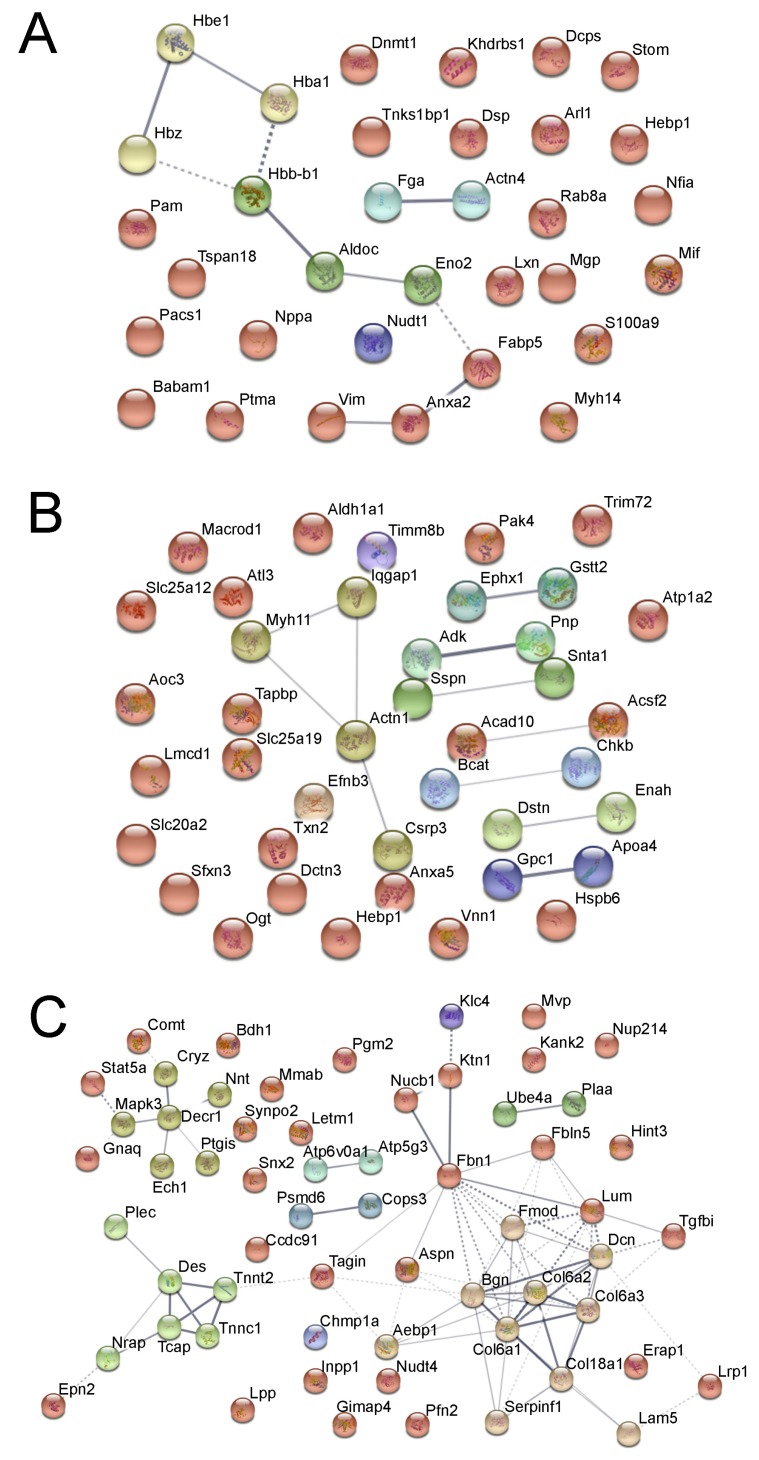
Fetal hypoxia-induced protein clustering in the heart. (**A**) Fetal heart. (**B**) Adult male heart. (**C**) Adult female heart. Each node represents a protein, edge between nodes shows known interactions. Color of the nodes shows different clusters, clustered by k-mean clustering algorithm.

In fetal heart samples, we detected 32 significantly regulated proteins (change > 15%, *p* < 0.05), of which all but one (S100a9) were downregulated by hypoxia. The most significantly enriched protein clusters are the “response to abiotic stimulus” (Actn4, Aldoc, Babam1, Dnmt1, Lxn, Mgp, Mif, Nppa, Pam, Vim) and globin proteins (Hbz, Hbe1, Hbb-b1, Hba2) (Figure 2A), showing the acute effect of hypoxia.

When we inspected the proteomics data in adult male hearts, we found 40 proteins that were significantly regulated by antenatal hypoxia (change >15%, *p* < 0.05), 18 were upregulated and 22 were downregulated. Within the 40 proteins, we found two significant protein clusters: (1) the Gstt2-Ephx1-Aldh1a1 cluster, which is involved in the cellular metabolic process; and (2) the Actn1-Myh11-Dstn-Iqgap1-Pak4 cluster, which is related to actin skeleton regulation (Figure 2B). 

The proteomics data in adult female hearts showed a large difference to the male heart. Protein quantitation among genders is far more prominent than treatment conditions. In total, we identified 60 significantly regulated proteins (change >15%, *p* < 0.05), all of which were downregulated in the hypoxic group. Within all 60 proteins, a significant portion was related to heart muscle development and function, including essential proteins for cardiovascular system development (Aebp1, Col6a3, Dcn, Lama5, Tagln, Tcap, Tnnc1, Tnnt2, Col18a1, Erap1, Fbn1, Gnaq, Lama5, Lrp1, Tgfbi, Des, Lum, Fmod, Bgn, Aspn) and myocardial collagens (Col18a1, Col6a1, Col6a2, Col6a3). The downregulation of many of the above-mentioned proteins are known to have negative effects on heart muscle function, e.g., lack of lumican (Lum) is known to cause abnormal collagens in mice heart tissue, with fewer and thicker fibril. Network analysis showed that the most enriched cellular component is within the cardiac troponin complex, especially in contractile fibers (Figure 2C). In signaling, the striated muscle contraction also showed significant enrichment (Tnnc1, Tnnt2, Tcap, Des). Gnaq and Mapk3 were found in “serotonin receptor 2 and ELK-SRF/GATA2” signaling pathway, one upstream and one downstream of calcium signaling. Moreover, we observed that a group of proteins related to mitochondria signaling and function was also significantly downregulated (Mapk3, Decr1, Ptgis, Cryz, Nnt). These findings suggest that antenatal hypoxia has a systematic suppressive effect on heart muscle development and function in female rats.

**Figure 3:**
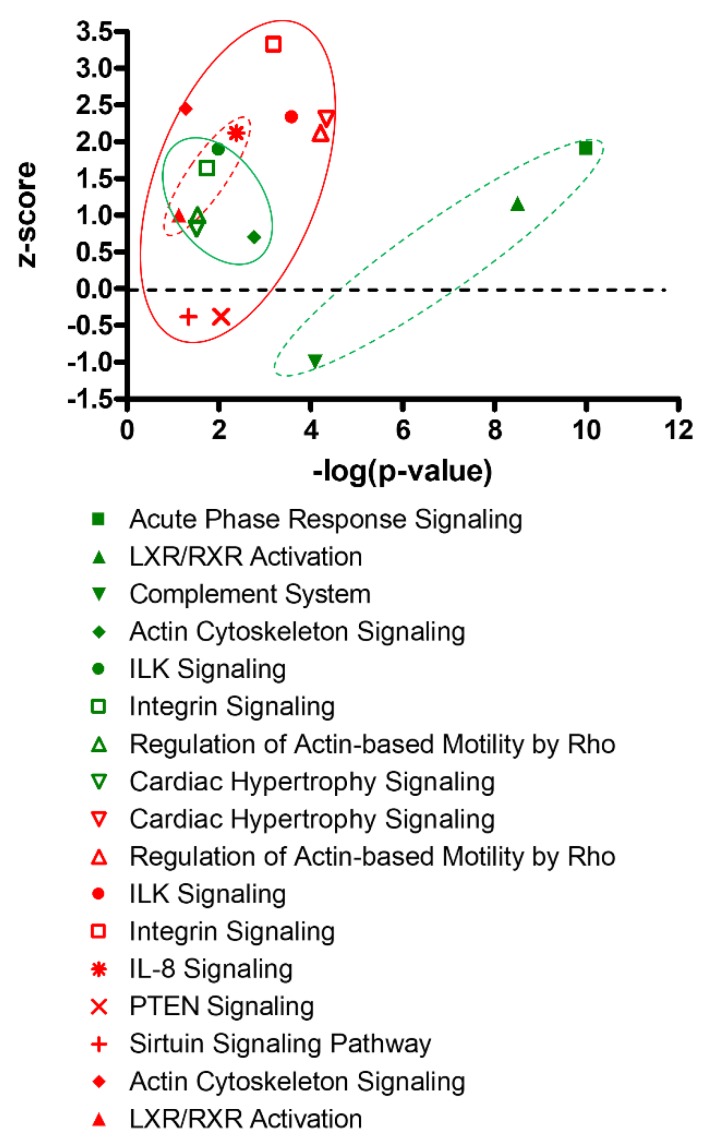
Canonical pathway analysis of fetal hypoxia-induced proteomics profiles in the heart. Green symbols: adult male heart; Red symbols: adult female heart; Circles with dot lines: immune/inflammatory related pathways; Circles with solid lines: myocyte growth/hypertrophy related pathways.

Canonical pathway analysis also revealed different patterns of pathway enrichment between male and female hearts, resulting from antenatal hypoxic treatment (Figure 3). In the male heart, prenatal hypoxia induced a significant enrichment of immune/inflammatory related pathways. In the female heart, the fetal hypoxia-mediated enrichment appeared more preferential in myocyte growth/hypertrophy related pathways. 

We further tested all identified proteins for their differential power to be used as potential biomarkers to differentiate gestational hypoxia and normoxic control. We tested all the possible combinations of 1–6 proteins using multivariate analysis of variance (MANOVA) and found a few groups of proteins with synergetic effects, which might be an indication of potential interaction. In fetal samples, proteins with most differential power were Nppa, Mif, Hbe1, Anxa2, Hba1, Hbb, S100a9, Ctnnb1, Actn4, and Fabp5, most of which are known to be involved in acute hypoxia. The human orthologues of these proteins are known to form three strong clusters (Supplementary Figure S3). Not surprisingly, the hemoglobin proteins are strong predictors of hypoxia. Interestingly, the combination of Anxa2 and Actn4 can be used to differentiate hypoxic and control conditions with a *p*-value < 0.00026, showing a significantly synergetic effect. In human, ANXA2 and ACTN4 are known to be co-expressed in genomic studies. However, the exact function of this combination is not clearly studied.

In female samples, proteins with the most differential powers were Psmd6, Bgn, Kank2, Mapk3, Nnt, Erap1, Col6a2, Ccdc91, Aspn, Aebp1, Ptgis, Serpinf1, Pfn2, Ube4a, Col18a1, and Epn2, the first seven proteins were especially powerful in differentiating gestational hypoxia and control samples. The human orthologues of these proteins belong to four clusters that are known for the involvement of muscle formation (Supplementary Figure S4). When combined and tested by MANOVA, several combinations of proteins showed significantly synergetic effects. These results showed that the long-term effects of gestational hypoxia on female hearts are mostly reflected on mitochondria, proteasome, and muscle development/function. The use of these biomarkers and combination of biomarkers can potentially allow us to diagnose individuals that have been exposed to hypoxia during early development.

In male samples, we found only seven proteins (Gpc1, Ogt, Gstt2, Vnn1, Hspb6, Pnp, Trim72) that can be used to differentiate gestational hypoxia from control group. Although no correlation was found among these proteins, they showed very strong synergetic effects as combinations of biomarkers (Supplementary Figure S5). Especially the combination of Gstt2, Vnn1, and Pnp gave a *p*-value of 5.01 × 10^−7^ after the MANOVA test. Interestingly, all three of them are enzymes (sulfatase, amidohydrolase, and phosphorylase), which are all involved in the TCA cycle and purine metabolism, consistent with the metabolomics data.

### 3.3. Effect of Antenatal Hypoxia on Metabolome in the Heart

**Figure 4:**
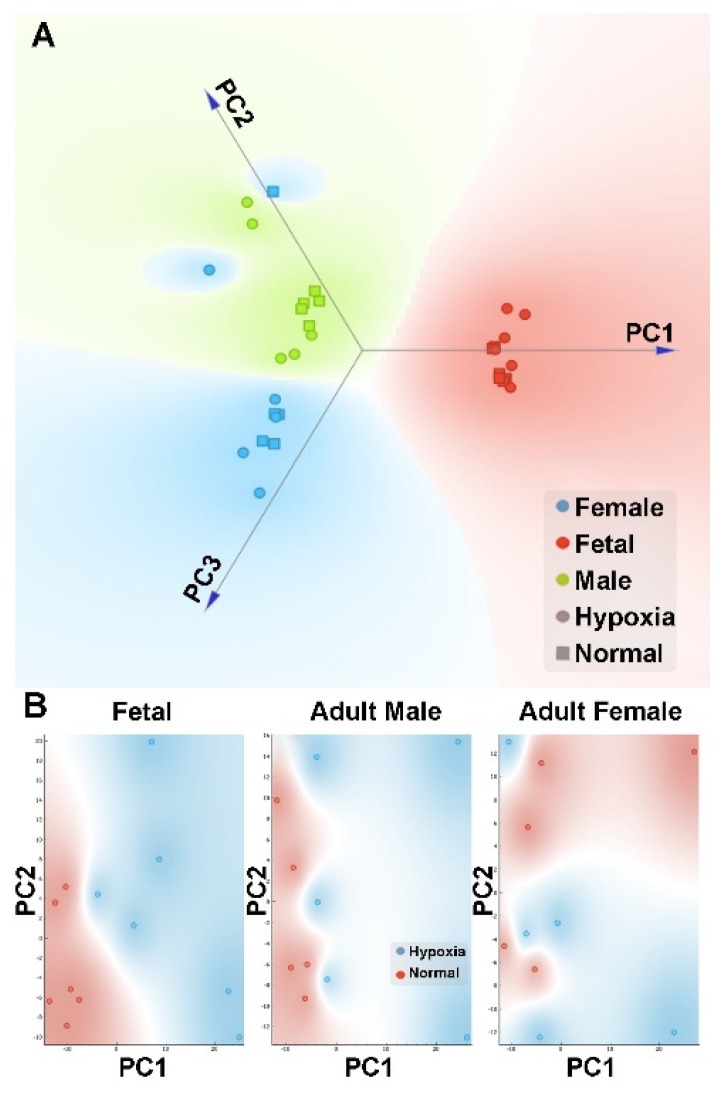
PCA plot of metabolomics data in the heart. Pregnant rats were treated with normoxia or hypoxia (10.5% O_2_) from day 15 to day 21 of gestation. Metabolomics profiling was determined in hearts isolated from near-term (21-day) fetuses and 5 month-old male and female offspring. (**A**) Linear projection of PC1 to PC3 of the PCA shows clear gender separation. Circle: hypoxia; Square: normal; Red: fetal; Blue: adult female; Green: adult male. (**B**) PC1 and PC2 plots of each gender shows that hypoxia (blue circle) samples can be clearly separated from normal (red circle) samples in fetal and adult male but not female.

Global metabolic profiles in the hearts were determined by Metabolon, Inc. In total, 603 metabolites were identified in fetal and adult cardiac tissues, of which 108 displayed statistically significant changes between fetal tissues exposed to normoxic or hypoxic conditions and 111 displayed statistically significant differences in adult tissues after exposure to these two conditions in utero (Supplementary Data 2). After normalization, we performed PCA analysis to the metabolomics data. We found that the gender effect was again a significant contributing factor in the differential metabolome in the heart (Figure 4A). We then analyzed each gender separately to explore the difference between hypoxic and normoxic treatment conditions. When analyzed within each gender, PCA analysis showed a clear difference in hypoxia and control in both fetal and male heart samples (Figure 4B). However, in female samples, there was a complicated pattern, possibly due to a large heterogeneity within the replicates. Of interest, there was a substantial sex dimorphic effect of fetal exposure to hypoxia with males demonstrating a markedly greater number of metabolic changes in adulthood than females. Specifically, 137 compounds reached statistical significance between hearts from male rats in the normoxic and hypoxia exposure groups whereas only 32 metabolites differed in adult females between the normoxic and hypoxic exposure groups. Comparisons between fetal and adult tissue groups showed statistical differences in approximately 80% of metabolites and reflect well-known and wide-ranging changes in metabolism between the fetal and adult state.

**Figure 5:**
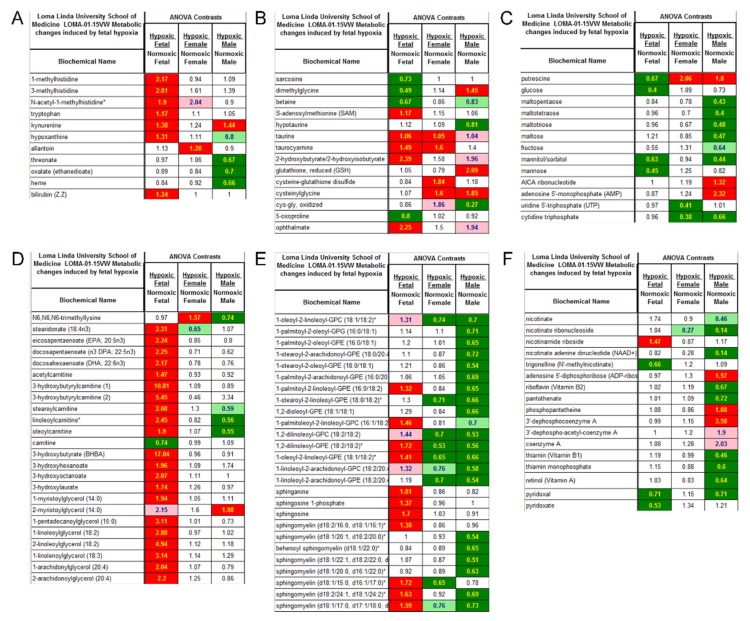
Pathway heat map of metabolomics data. (**A**) Inflammation and oxidative stress; (**B**) sulfur amino acids, methylation, and oxidative stress; (**C**) glycogen metabolism; (**D**) fatty acid oxidation; (**E**) membrane complex lipids; (**F**) cofactors. Green: indicates significant difference (*p* < 0.05) between the groups shown, metabolite ratio of <1.00; Light green: narrowly missed statistical cutoff for significance 0.05 < *p* < 0.10, metabolite ratio of <1.00; Red: indicates significant difference (*p* < 0.05) between the groups shown; metabolite ratio of ≥1.00; Light red: narrowly missed statistical cutoff for significance 0.05 < *p* < 0.10, metabolite ratio of ≥1.00; Non-colored text and cell: mean values are not significantly different for that comparison.

Figure 5 shows pathway heat map of metabolomics data. Fetal heart tissues from the hypoxia treatment group showed elevations in stress markers associated with muscle breakdown, inflammation, oxidative stress, and red blood cell turnover. The elevation of these markers only partially persisted into adulthood (Figure 5A, Supplementary Figure S6). Biomarkers associated with sulfur amino acids, such as methionine and cysteine, important for methylation, antioxidants, and glutathione were altered in hypoxic fetal hearts and greater oxidative stress was evident in cardiac tissue of adults exposed to hypoxia in utero (Figure 5B, Supplementary Figure S7). Maltose and maltooligosaccharides, markers of glycogen metabolism, were significantly decreased in hearts of male rats exposed to hypoxia in utero whereas metabolic activators of AMP kinase (AMPK) activators, AICA ribonucleotide and AMP, were elevated (Figure 5C, Supplementary Figure S8). The elevation of monoacylglycerols, acyl-carnitines, 3-hydroxy-acids, and 3-hydroxybutyrate in hypoxic relative to normoxic fetal cardiac tissue depicted a greater level of fatty acid oxidation under hypoxia. In fetal heart samples, we noticed a significant regulation of ketone metabolism, indicated by an elevated level of hydroxyalkylnoate, such as hydroxybutanoate, hydroxyhexanoate, hydroxyoctanoate, and hydroxy-2-ethyl-propionate. Long-chain acyl-carnitines and the carnitine precursor N6-trimethyllysine were significantly diminished in hearts from male adult rats exposed to hypoxia in utero but most other markers of fatty acid oxidation did not differ between the adults from the normoxic and hypoxic in utero groups (Figure 5D, Supplementary Figure S9). In addition, exposure to hypoxia in utero appeared to have long-term effects on membrane lipid composition and levels (Figure 5E, Supplementary Figure S10). Furthermore, cardiac tissue from adult male rats exposed to hypoxia in utero displayed alterations in the levels of a number of cofactors that could have an impact of NAD+ and coenzyme A for energy metabolism and thiamin and pyridoxal groups for proper enzyme function (Figure 5F, Supplementary Figure S11).

Metabolic pathway enrichment analysis showed that “protein biosynthesis”, “TCA cycle”, “purine metabolism” and “arginine and proline metabolism” pathways were significantly regulated by antenatal hypoxia in the hearts of all three groups (Supplementary Figures S12–S14). 

### 3.4. Integrated Multi-Omics Data Analysis

To better understand the underlying molecular mechanism and to trace the source of the observed gene/protein changes between hypoxia and control groups in the heart, we integrated our transcriptomics data, proteomics data, and metabolomics data together using an in-house informatics pipeline (Supplementary Figure S15). RNA-seq data were obtained from the previous publication in the same treatment groups and the same tissues [26], assembled using NCBI rat reference genome and analyzed using R packages EdgeR for differential expression analysis (Supplementary Figure S16). PCA of transcriptomics data showed that fetal samples were clearly separated from the rest while adult male and female samples were also differentiable (Supplementary Figure S17). 

In a typical proteomics experiment, the existence of proteins is not known until identified by comparing mass spectrum with reference protein database. In standard reference proteome databases, all the proteins related to rat are included, despite the strain or condition applied. Many proteins and protein isoforms, although lack transcripts level evidence, are often used in protein identification and thus result in a significant loss of statistical power in the estimation of protein false discovery rate. [31] In this study, transcriptomics was performed on the same sample and therefore provided transcript level evidence to support the removal of unrelated protein from reference database. To improve the quality of proteomics data, we used the transcriptomics data to construct a custom reference proteome database for this data set, which only included proteins and protein isoforms with transcripts level evidence. Specifically, we first combined all the assembly files and then mapped them to NCBI rat reference genome. We then performed a six-frame in silico translation to generate all the possible protein sequences. To improve proteomics data quality and facilitate downstream data analysis with existing protein interaction networks and ontology annotations, we excluded protein sequences that were not currently known in our protein interaction/annotation databases. All the in silico generated protein sequences were cross referenced with Swissprot rat reference proteome and all the proteins without any transcripts level evidence were removed to form a custom reference proteome database. 

The goal of integrated multi-omics data analysis is to treat each omics data set as an independent observation of the same biological system at different levels. After integration, we would be able to cross-validate the significance of our findings among all three omics data sets and therefore differentiate the true signals from noise. In order to integrate all the data, we chose to map all the data to genes and then gene clusters using existing metabolic pathway and protein complex information from the database. Here, a gene cluster is defined as a group of genes that are similar in sequence and function, as defined in KEGG SSDB (Sequence Similarity DataBase) Specifically, for each identified metabolite, KEGG and Reactome pathways are used to map metabolite to corresponding pathways. Each pathway is then mapped to all the corresponding proteins and genes; proteomics data are mapped to the corresponding protein complexes and genes; transcriptomics data are mapped to gene clusters. We then calculated a combined probability score using Fisher’s combined probability test. Specifically, for each pathway, we first calculated the *p*-values using transcriptomics, proteomics, or metabolomics data respectively using the previously described method. [32] All the *p*-values are then integrated using Fisher’s method. 

The scores of each pathway were then compared and ranked by dynamic programming to remove repetitive use of the same data point and to find the best solution that can explain the overall data trend. Here we used dynamic programming to limit the number of assignments of each metabolite/protein/gene without significantly affecting the overall significance level. This step helped us to eliminate a significant amount of noise/outliers from the original data.

**Figure 6:**
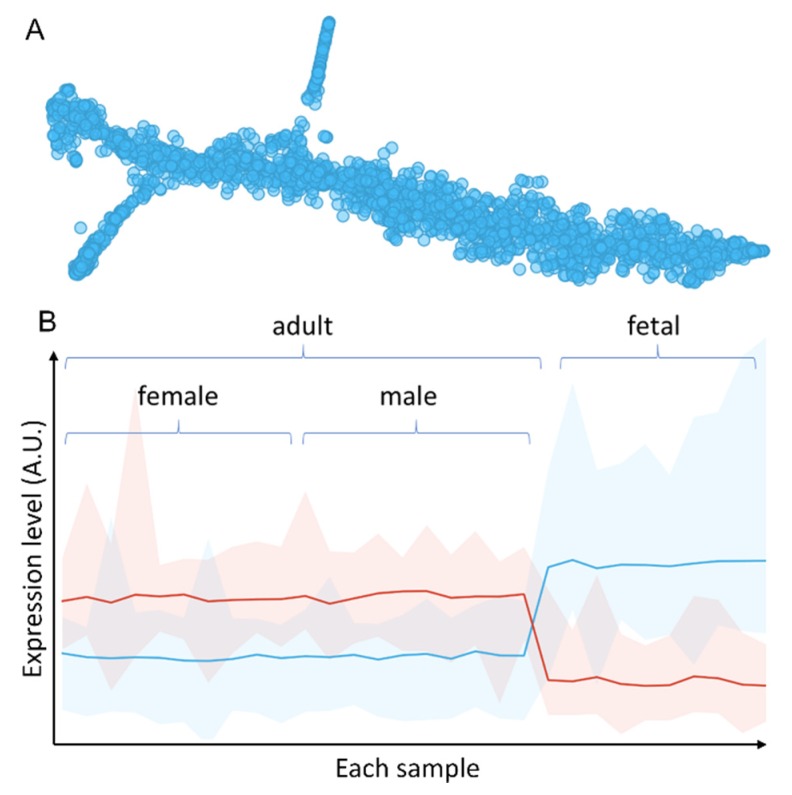
Integrated analysis of multi-omics data. (**A**) t-SNE clustering of integrated multi-omics data shows two clusters that separated from the main data stream, the upper middle cluster and the lower left cluster. (**B**) Overall expression levels of gene/protein group 1 of the upper middle cluster (blue) and group 2 of the lower left cluster (red). X-axis shows the samples. Y-axis shows expression level.

In order to find persistent changes caused by antenatal hypoxia, i.e., common pathways that were affected by antenatal hypoxia and lasted till adulthood, we used data from all genders and performed unbiased clustering analysis. Comparing to conventional analysis approach, which consists of data filtering by fold changes and *p*-values, then followed by enrichment analysis of the filtered genes within each gender, our approach utilized data points from three omics data sets and three genders, including those that would not pass significant test in any individual omics data set or gender group. Each gene/protein was then clustered using t-Distributed Stochastic Neighbor Embedding (t-SNE). As shown in Figure 6A, after clustering there were two clusters that separated from the main data stream, the upper middle cluster and the lower left cluster. The upper middle cluster, named group 1, was significantly upregulated in fetal samples comparing to adult rats whereas the group 2 of the lower left cluster, was significantly downregulated in the fetal heart (Figure 6B). Analysis of the genes/proteins in the two clusters revealed that they mainly belonged to the mitochondria, with a GO cellular component enrichment FDR of 3.99 × 10^−122^, identifying 123 of 817 all known mitochondrion related proteins, and 94 of 514 mitochondrial part proteins. The top enrichment results were shown in Table 1. We then mapped these identified genes together with other genes in a volcano plot, we found these genes mainly distributed in the variable regions (far left and far right) of the plot, showing significant changes in all three gender groups (Supplementary Figure S24). These results confirmed that these changes are persistent throughout the development of the rats. We have noted that many of the genes identified by this approach would not pass the standard statistical test within each individual gender group. The integration of data from all genders and multi-omics allowed us to scavenge some of less confident data points that would have been missed by the conventional approach. From the enrichment results, it is very clear that mitochondria play a vital role in both short and long-term effects of antenatal hypoxia. Next, we analyzed all the genes (189 in total) within group 1 and group 2 clusters (Supplementary Figures S18–S20), and tried to identify the key genes that dictate the long-term hypoxia effects in adult rats. We found some genes that could be used to differentiate both the male and female rats between hypoxia and control groups. These common genes include Ndufs3, Ndufv2, Ndufs4, Ndufb5, and LOC100912599 from the Complex I, as well as other electron transport chain related genes such as Acsf2, Adhfe1, Atp5g3, Atp5e, Bcat2, Dnaja3, Sod2, Ak3, Ivd, and Fabp3. Mitochondria Complex I is one of the most crucial complexes in the respiratory chain, especially in heart tissues, and it is a very stable complex with a low turnover rate. When we correlate these proteins to previously published protein turnover rate data [33], we found that many of these proteins have a very long half-life comparing to other cytosolic or membrane proteins. On average, the Complex I proteins identified from our analysis have a three times slower turnover rate comparing to cytosolic proteins. 

We next examined the protein/gene changes unique to male hearts and found them mainly in the metabolic process and TCA cycle, namely Atp5s, Mdh1, Aco2, Pfkm, Aldh4a1, Aldh2, Mecr, Acad9, and Pdk1. In female hearts, the main differentially expressed proteins/genes within group 1/group 2 clusters were the troponin family including Tnnc1, Tnnt2, and Tnni3, as well as other enzymes such as Myl2, Cox5a, and Acot9. This result shows that in female hearts, antenatal hypoxia resulted in an elevated level of troponin and striated muscle, possible due to a compensation mechanism. While in the male heart, the change is mainly metabolic, including an elevated level of enzyme activity in the hypoxia group.

## 4. Discussion

Gestational hypoxia has adverse impact in developmental plasticity and increases the risk of cardiac dysfunction and ischemic heart disease, among others, in offspring [5]. Recently, we demonstrated that antenatal hypoxic stress altered global DNA methylation patterns and resulted in epigenetic reprogramming of the transcriptome in the heart [26]. In the present study, we provided further novel insights into phenotypic reprogramming of proteome and metabolome in the heart, resulting from antenatal hypoxia-induced epigenotype. In general, in response to fetal hypoxia there were differential phenotypic changes in proteomics and metabolomics profiling between the fetal heart and adult heart, as well as between male and female adult hearts. Of importance, the unbiased assessment of multi-omics integration across the transcriptome, proteome, and metabolome revealed the major effect of antenatal hypoxia on the mitochondrion in the heart, providing new insights into phenotypic reprogramming of the mitochondrion in the heart and mechanistic understanding of fetal stress-induced programming of heart disease in adulthood. 

Comparing the fetal hearts between hypoxia and control groups, we found that gestational hypoxia resulted in an elevated level of multiple gene/protein clusters in the heart, including hemoglobin upregulation and systematic response to abiotic stimulus/stress, all of which are previously known as acute responses to hypoxia. Stress-related metabolites were also significantly upregulated, showing significant upregulation of the alternative metabolic pathways for survival. The comparison between fetal and adult hearts showed that the long-term effects of antenatal hypoxia on the proteome are vastly different than the immediate effects. This is consistent with our previous findings showing that gestational hypoxia epigenetically regulated a distinct set of genes in the fetal and adult hearts [26]. In the present study, the proteomics data showed that the long-term disturbance of the proteome might be mediated by the abnormal development of the cardiovascular system, especially within the cardiac muscle system. It is possible that the differences in proteome change between adult male and female hearts were caused by the estrogen-related proliferative response of cardiac fibroblasts to hypoxia [34]. The proteomics data alone, however, do not show a clearly shared pattern in adult hearts, between the ones exposed to prenatal hypoxia and controls. The identified gene/protein clusters from male hearts are related to cellular metabolic process and actin skeleton regulation while the clusters identified from female hearts are more related to cardiovascular system development and myocardiac collagen formation. Canonical pathway analysis of proteomics data also revealed very different patterns of pathway enrichment between male and female hearts, resulting from antenatal hypoxic treatment. Fetal hypoxia induced a significant enrichment of immune/inflammatory related pathways in the male heart, yet it appeared to have a more preferential effect on myocyte growth/hypertrophy related pathways in the female heart. Consistent with this finding, we previously demonstrated that fetal hypoxia induced reprogramming of transcriptome with a significant enrichment of signal pathways in pro-inflammatory/immune response in the heart of offspring, which was significantly greater in the heart of adult male than female [26]. 

In agreement with the findings of proteomics data, analysis of metabolomics profiling revealed a substantial dimorphic effect of fetal exposure to hypoxia with male hearts demonstrating a markedly greater number of metabolic changes in adulthood than female hearts. Specifically, 137 compounds reached statistical significance between hearts from male rats in the normoxic and hypoxia exposure groups whereas only 32 metabolites differed in adult females between the normoxic and hypoxic exposure groups. This suggests that females were more resilient and/or that males were more sensitive in the response, recovery, and reprogramming of metabolomics profiles to hypoxia in utero. In addition to sex differences, fetal and adult hearts showed significant differences in approximately 80% of metabolites and reflected well-known and wide-ranging changes in metabolism between the fetal and adult state. Fetal hearts from the hypoxia treatment group showed elevations in stress markers associated with muscle breakdown, inflammation, oxidative stress, and red blood cell turnover, some of which persisted into adulthood. The finding of increased 1-methylhistidine and 3-methylhistidine suggested a higher level of muscle protein breakdown in hypoxic fetal hearts. Although these changes were not found in adult hearts, the elevation of kynurenine, an inflammatory stress marker derived from tryptophan, in the hypoxic fetal heart persisted into adult male hearts. This is consistent with the findings of proteomics analysis showing the enrichment of immune/inflammatory related pathways in the male heart. Elevated levels of heme-derived bilirubin in hypoxic fetal tissue suggested a sign of red blood cell damage or an increased demand for this endogenous antioxidant. Hypoxic adult hearts also showed signs of increased oxidative stress. This was manifested in adult female hearts by an elevation of allantoin, which is an oxidative derivative of the endogenous antioxidant urate. In the male heart, a significant decrease of oxidative derivatives of ascorbate was detected. Altogether, these findings revealed an acute state of metabolic stress and cell dysfunction or death in hypoxic fetal hearts and, perhaps, a modest level of inflammatory or oxidative stress persisting into adulthood.

Alterations in methionine and cysteine metabolism in hypoxic fetal hearts showed some penetration to the metabolic phenotype of adult hypoxic hearts. The finding of accumulation of the methionine-derived universal methyl donor S-adenosylmethionine and subsequent decrease in markers of methionine regeneration, betaine and dimethylglycine, suggested methylation-dependent reactions and epigenetic modifications in the heart. This is consistent with the previous study showing that fetal hypoxia had a significant effect on the methylome and reprogramming of transcriptome in the heart [26]. Cysteine metabolism leading to the non-proteogenic amino acid taurine was elevated in hypoxic tissues from fetal and adult hearts and a derivative of taurine, taurocyamine also known as GES, was elevated as well. Taurine may act as an antioxidant in response to increased oxidative stress in the heart. Interestingly, taurocyamine is an antagonist of taurine transporter and it causes decreased contractile function of skeletal and cardiac muscle [35,36]. Cysteine also provides the essential reactive thiol group for glutathione and multiple markers of glutathione oxidation and turnover, Cys-glutathione disulfide, Cys-Gly (reduced), and Cys-Gly (oxidized), showed changes perhaps indicative of greater oxidative stress in adult female hypoxic hearts, whereas elevations of 2-hydroxybutyrate that cannot be distinguished from a typically minor compound 2-hydroxyisobutyrate and ophthalmate in hypoxic fetal heart samples potentially portrayed insulin resistance and depletion of glutathione [37,38]. 

Metabolomics profiling also revealed an elevation of fatty acid oxidation in fetal hypoxic heart tissue and altered glycogen metabolism in hypoxia-exposed male hearts. The elevation of monoacylglycerols, acyl-carnitines, 3-hydroxy-acids, and 3-hydroxybutyrate in hypoxic relative to normoxic fetal cardiac tissues depicted a greater level of fatty acid oxidation under hypoxia. Long-chain acyl-carnitines and the carnitine precursor N6-trimethyllysine were significantly diminished in hearts from male adult rats exposed to hypoxia in utero but most other markers of fatty acid oxidation did not differ between the adults from the normoxic and hypoxic in utero groups. Markers of glycogen metabolism, maltose and maltooligosaccharides were significantly decreased in hearts of male rats exposed to hypoxia in utero whereas metabolic activators of AMP kinase (AMPK) activators, AICA ribonucleotide and AMP, were elevated. Although acute activation of AMPK during energy stress inhibits glycogen synthesis, chronic activation paradoxically leads to glycogen accumulation in the heart [39]. Cardiac AMPK influences many physiological and pathophysiological processes in the heart including maintaining energy balance, glycogen accumulation, and hypertrophy [40]. Although ATP was not detected, the increase of AMP and decrease of nucleotide triphosphates, such as CTP and UTP, was a modest sign of energy stress in adult hypoxic tissues. Given that polyamine production is often highly correlated with cell growth, the finding that the polyamine putrescine was significantly decreased in hypoxia fetal heart tissue but significantly elevated in hypoxia adult heart tissue from both genders is intriguing and suggests hypertrophy in adult hearts from rats exposed to hypoxia in utero. This is again consistent with proteomics data showing the enrichment of myocyte growth/hypertrophy related pathways in the adult hearts. 

In addition, exposure to hypoxia in utero appeared to have long-term effects on membrane lipid composition and levels in both male and female hearts. The general trend was for glycerophospholipids, such as 18:2/18:2-GPC and 18:2/18:2-GPE, and sphingomyelins to be elevated in hypoxic fetal hearts but decreased in hypoxic adult hearts, especially in males. Since phospholipids and sphingomyelins are essential for cellular membranes and cellular function, these changes could impact heart health and performance [41]. In addition to sphingomyelins that serve as membrane components, sphingosine and sphingosine-1-phosphate (S1P) were elevated in hypoxic fetal hearts. This may be important because S1P has cardioprotective effects [42,43], and this may be yet another hypoxia-induced stress response. The finding that fetal hypoxia altered the levels of a number of cofactors that impact NAD+ and coenzyme A for energy metabolism and thiamin and pyridoxal groups for proper enzyme function in the male heart is interesting. Because these cofactors are dietary micronutrients, their changes may point to long-term effects of antenatal hypoxia on nutrient absorption in male hearts.

Multi-omics integration of transcriptome, proteome, and metabolome data provides a powerful means to understand and accurately predict biological behavior in a given condition, and this approach has recently been implicated in several research areas of cancer metabolism [44,45] and microbiology [46,47]. To reduce the complexity of a vast amount of data in multi-omics integration, we used multiple mathematical approaches in the present study, including t-distributed stochastic neighbor embedding (t-SNE) to isolate the two main clusters; fast independent component analysis (ICA) to decompose modules from the clusters; Fisher’s combined probability test to integrate multi-omics data; and MANOVA to differentiate combinations of elements that are most effective in differentiating samples. Thus, the multi-omics integration in the present study significantly improved data analysis in three aspects: (1) cross-validation; (2) fight against noise to improved confidence; and (3) fill in the missing components. By integrating multi-omics data, the unbiased evidence leads us to identify the major target of the mitochondria in fetal hypoxia-induced phenotypic reprogramming in the heart. The confidence in identifying genes/protein clusters and metabolic pathways in the mitochondria was vastly enhanced by multi-omics integration, with a GO cellular component enrichment FDR of 3.99 × 10^−122^, identifying 123 of 817 all known mitochondrion related protein, and 94 of 514 mitochondrial part proteins. Further analysis revealed that within the respiratory chain of mitochondria, multiple subunits of Complex I, including many lipid processing enzymes were significantly altered in hypoxia groups, suggesting that Complex I is the main target that is responsible for changes induced by hypoxia. Metabolomics data also confirmed that the metabolic functions around the TCA cycle is altered by hypoxia in the adult heart. Mitochondria Complex I is one of the most crucial complexes in the respiratory chain, especially in the heart. It is also a very stable complex with a low turnover rate. Given the slow turnover rate of the proteins in Complex I, it is possible that some early assembly errors in the heart caused by gestational hypoxia persist into adulthood and impact mitochondrial function and metabolic process and cardiac muscle development. The integrated multi-omics data also suggest the long-term effects of gestational hypoxia are different between the adult male and female hearts. The key differences are focused on a few gene/protein clusters. The most significant clusters in adult male rats were found within the cellular metabolic process and actin skeleton regulation. Both proteomics and metabolomics evidence implied the metabolic effects to be related to purine metabolism and TCA cycle. In contrast, a milder and more systematic suppressive effect was observed in the development of the cardiovascular system and myocardial collagens on female adult rats with gestational hypoxia.

Given that the mitochondrion plays a pivotal role in energy metabolism, oxidative stress, inflammation and apoptosis, particularly in the heart, the hypoxia-induced phenotypic reprogramming of the mitochondrion in the heart is likely to have a significant impact in heart function and vulnerability to disease. In the same animal model, our previous studies demonstrated that fetal hypoxia caused a decrease in cardiomyocyte proliferation and premature exit of the cell cycle of cardiomyocytes, leading to fewer but larger myocytes in the heart later in life [18,48,49,50,51]. Of importance, antenatal hypoxia resulted in pathological cardiac remodeling with left ventricular hypertrophy and an increase in heart susceptibility to ischemia and reperfusion injury in the adult heart [17,19,21,22,23,24,52]. In addition, gestational hypoxia altered mitochondrial protein expression and decreased cytochrome c oxidase activity in the fetal heart, which persisted into the adult heart [53,54,55]. The altered mitochondrial proteins and function resulted in a mismatch between myocardial glycolysis and glucose oxidation rates and an increase in myocardial production of acetyl-CoA during reperfusion after ischemia, and this mismatch between energy production and cardiac performance contributed to an increased susceptibility of the heart to ischemia and reperfusion injury observed in the adult heart resulting from fetal hypoxia [52,56].

Overall, the present study demonstrated that gestational hypoxia not only altered proteomics and metabolomics profiling in the fetal heart, but also it had a long-lasting impact on the heart after birth in adulthood. In addition, the effects were developmental stage and gender dependent. Of importance, multi-omics integration revealed novel insights into fetal stress-induced phenotypic reprogramming of the mitochondrion in the heart, impacting cardiac energy metabolism, oxidative stress, inflammation and apoptosis and linking antenatal hypoxia and increased heart susceptibility to disease later in life. The unbiased assessment of multi-omics integration and the findings of fetal hypoxia-induced differential changes in proteomics and metabolomics profiling in the heart at different developmental stages and sexes in the present study provide a very useful database for future investigations of phenotypic alterations in the heart under physiological or pathophysiological conditions. 

## Figures and Tables

**Table 1:** Top enriched pathway/gene clusters for group1/2 genes identified from t-SNE.

Pathway	Count in Gene Set	FDR
**Biological Process (GO)**		
small molecule metabolic process (GO:0044281)	102/933	1.94 × 10^−83^
oxidation-reduction process (GO:0055114)	82/564	1.06 × 10^−73^
generation of precursor metabolites and energy (GO:0006091)	50/187	4.30 ×10^−54^
**Molecular Function (GO)**		
catalytic activity (GO:0003824)	118/2668	3.23 × 10^−58^
oxidoreductase activity (GO:0016491)	61/458	3.20 × 10^−51^
cofactor binding (GO:0048037)	46/350	1.55 × 10^−37^
**Cellular Component (GO)**		
mitochondrion (GO:0005739)	123/817	3.99 × 10^−122^
mitochondrial part (GO:0044429)	94/514	1.14 × 10^−95^
mitochondrial matrix (GO:0005759)	52/179	7.22 × 10^−59^
**KEGG Pathway**		
metabolic pathways (rno01100)	109/1240	3.34 × 10^−82^
oxidative phosphorylation (rno00190)	40/130	1.52 × 10^−45^
carbon metabolism (rno01200)	37/112	4.08 × 10^−43^
**Reactome Pathway**		
TCA cycle and respiratory electron transport (RNO-1428517)	46/123	8.62 × 10^−56^
metabolism (RNO-1430728)	82/1330	2.63 × 10^−47^
pyruvate metabolism and TCA cycle (RNO-71406)	22/34	2.13 × 10^−30^

## Supplementary Materials

The following are available online at https://www.mdpi.com/2073-4409/8/12/1608/s1, Figure S1: Overall Study Design, Figure S2: K-mean clustering analysis of gene contribution to PC. Figure S3: Protein clustering of the most differentiable proteins for hypoxia vs. normoxia in the fetal heart, based on known interactions of human orthologues. Figure S4: Protein clustering of the most differentiable proteins for hypoxia vs. normoxia in the adult female heart, based on known interactions of human orthologues. Figure S5: Protein clustering of the most differentiable proteins for hypoxia vs. normoxia in the adult male heart, based on known interactions of human orthologues. Figure S6: Metabolites of inflammation and oxidative stress pathways. Figure S7: Metabolites of sulfur amino acids, methylation, and oxidative stress pathways. Figure S8: Metabolites of glycogen metabolism pathways. Figure S9: Metabolites of fatty acid oxidation pathways. Figure S10: Metabolites of membrane complex lipids pathways. Figure S11: Metabolites of cofactor pathways. Figure S12: Metabolic pathway enrichment analysis in the fetal heart. Figure S13: Metabolic pathway enrichment analysis in the adult male heart. Figure S14: Metabolic pathway enrichment analysis in the adult female heart. Figure S15: Multi-omics data integration strategy to calculate the combined pathway enrichment score. Figure S16: Raw RNA-seq expression heatmap without and with K-mean clustering. Figure S17: Projection of PC1-3 of the PCA of RNA-seq expression data. Fetal heart, male and female samples are clearly separated by PC1-3. Figure S18: Clustering of all 189 identified genes from t-SNE mitochondria cluster. Figure S19: Groups 1 and 2 genes overview in all samples. Figure S20: (A) Group 1 genes in the fetal heart. (B) Group 2 genes in the fetal heart. (C) Group 1 genes in the male heart. (D) Group 2 genes in the male heart. (E) Group 1 genes in the female heart. (F) Group 2 genes in the female heart. Figure S21: Functional network enrichment analysis using (A) mouse orthologues or (B) human orthologues. Figure S22: Top 20 pathways identified from functional network enrichment analysis. Figure S23: Comparison of identified genes from functional network enrichment analysis using (A) mouse orthologues or (B) human orthologues. Figure S24: Volcano plot of (A) adult female rat (B) adult male rat (C) fetal rat samples using the log_2_(fold change) between control and hypoxia group as x-axis and -log_10_(p-value) as y-axis, Data 1: Protein identification and quantitation across all biological samples and conditions; Data 2: Metabolites identification and quantitation across all biological samples and conditions.
